# Formation of Rigid, Non-Flight Forewings (Elytra) of a Beetle Requires Two Major Cuticular Proteins

**DOI:** 10.1371/journal.pgen.1002682

**Published:** 2012-04-26

**Authors:** Yasuyuki Arakane, Joseph Lomakin, Stevin H. Gehrke, Yasuaki Hiromasa, John M. Tomich, Subbaratnam Muthukrishnan, Richard W. Beeman, Karl J. Kramer, Michael R. Kanost

**Affiliations:** 1 Department of Biochemistry, Kansas State University, Manhattan, Kansas, United States of America; 2 Division of Plant Biotechnology, College of Agriculture and Life Science, Chonnam National University, Gwangju, Korea; 3 Department of Chemical and Petroleum Engineering, University of Kansas, Lawrence, Kansas, United States of America; 4 Center for Grain and Animal Health Research, Agricultural Research Service, United States Department of Agriculture, Manhattan, Kansas, United States of America; Janelia Farm Research Campus, Howard Hughes Medical Institute, United States of America

## Abstract

Insect cuticle is composed primarily of chitin and structural proteins. To study the function of structural cuticular proteins, we focused on the proteins present in elytra (modified forewings that become highly sclerotized and pigmented covers for the hindwings) of the red flour beetle, *Tribolium castaneum*. We identified two highly abundant proteins, TcCPR27 (10 kDa) and TcCPR18 (20 kDa), which are also present in pronotum and ventral abdominal cuticles. Both are members of the Rebers and Riddiford family of cuticular proteins and contain RR2 motifs. Transcripts for both genes dramatically increase in abundance at the pharate adult stage and then decline quickly thereafter. Injection of specific double-stranded RNAs for each gene into penultimate or last instar larvae had no effect on larval–larval, larval–pupal, or pupal–adult molting. The elytra of the resulting adults, however, were shorter, wrinkled, warped, fenestrated, and less rigid than those from control insects. TcCPR27-deficient insects could not fold their hindwings properly and died prematurely approximately one week after eclosion, probably because of dehydration. TcCPR18-deficient insects exhibited a similar but less dramatic phenotype. Immunolocalization studies confirmed the presence of TcCPR27 in the elytral cuticle. These results demonstrate that TcCPR27 and TcCPR18 are major structural proteins in the rigid elytral, dorsal thoracic, and ventral abdominal cuticles of the red flour beetle, and that both proteins are required for morphogenesis of the beetle's elytra.

## Author Summary

Primitive insects have two pairs of membranous flight wings, but during the evolution of the beetle lineage the forewings lost their flight function and became modified as hard, rigid covers called elytra for protection of soft body parts of the abdomen and also the delicate flexible hindwings, which retained their flight function. This transformation is manifested by a greatly thickened and rigid (sclerotized) exoskeletal cuticle secreted by the forewing epidermis. We demonstrate that this evolutionary modification is accompanied by the incorporation of two highly abundant structural proteins into the elytral cuticle, namely TcCPR18 and TcCPR27. Depletion of these proteins by RNA interference results in malformation and weakening of the elytra, culminating in insect death. These proteins are also abundant in hard cuticle from other regions such as the pronotum and ventral abdomen, but are absent in soft cuticles, and therefore may function as key determinants of rigid cuticle. Expression of such proteins at high levels in the modified forewing appears to have been a fundamental evolutionary step in the transformation of the membranous wing into a thickened and rigid elytron in the Coleoptera.

## Introduction

How arthropods manufacture exoskeletons with a wide array of mechanical properties, ranging from hard and rigid to soft and flexible, is an important question in developmental biology. The insect exoskeleton, or cuticle, covers the entire body wall and attached appendages as well as the foregut, hindgut and tracheae. It is a complex extracellular biocomposite, secreted by the epidermis and consisting of several functional layers including the waterproofing envelope, the protein-rich epicuticle and the chitin-rich procuticle [1]. Cuticular proteins (CPs) and the polysaccharide chitin are the primary structural components of the exo- and endocuticular layers that comprise the procuticle. During cuticle maturation and tanning (sclerotization and pigmentation), some of the CPs are post-translationally modified and cross-linked by quinones or quinone methides produced by the laccase-mediated oxidation of N-acylcatechols [2], [3]. This process stabilizes and hardens the exoskeleton, protecting insects from microbial, physical and other environmental stresses. However, little is known about the functional importance of individual insect cuticular proteins in the morphogenesis and mechanical properties of the exoskeleton.

More than 100 genes encoding CP-like proteins have been identified in the fruit fly, *Drosophila melanogaster* [4], with a similar number present in the red flour beetle, *Tribolium castaneum* [5]. *Anopheles gambiae* (malaria mosquito) and *Bombyx mori* (oriental silkworm) have an even larger number of genes encoding CP-like proteins, each species harboring more than 200 putative *CP* genes [6]–[10]. Expression of specific CPs may be required to produce cuticles with a range of morphological and mechanical properties in different regions of the insect body and at different developmental stages.

Insect CPs are classified into several distinct families defined by the presence of specific sequence motifs [7], [10]. The largest of these is the CPR family, which includes proteins that have a conserved amino acid sequence known as the Rebers & Riddiford (R&R) motif [11]. The R&R motif contains a putative chitin-binding domain that may help to coordinate the interactions between chitin fibers and the proteinaceous matrix [12], [13].

A major event in the evolution and diversification of beetles was the transformation of the membranous forewings into thickened, hardened, non-flight covers (elytra) for protection of the delicate hindwings and dorsal abdomen [14]–[16]. The elytron is composed of ventral and dorsal layers of epidermal cells that secrete thin lower and thicker upper cuticular laminae [17], [18]. In the developing elytron, the space between these two layers is filled with hemolymph and supporting structures known as trabeculae that function as mechanical struts, connecting and fortifying the dorsal and ventral cuticular layers. As the elytron matures, the epidermal layers are reduced in size, possibly dying or fusing together, and the hemolymph is resorbed, leaving a cavernous interior. The dorsal layer of the elytron becomes highly tanned and rigid as a result of both pigmentation and sclerotization. The ventral layer also exhibits some pigmentation, but it remains thin and membranous in comparison to the dorsal layer. The surface of the ventral elytral cuticle is relatively smooth and makes close contact with the underlying and folded hindwings. The surface of the dorsal elytral cuticle, on the other hand, contains numerous sensory setae and rib-like structures (striae) that extend longitudinally, apparently adding rigidity to the structure [19]. Initially, the elytra are short, colorless and soft, but they expand in both length and width shortly after eclosion, and subsequently harden and darken. A similar cuticle tanning process occurs in most of the adult body wall.

In this study we have identified two highly abundant proteins that are present in rigid cuticle of the elytron, pronotum and ventral abdomen but not in the flexible cuticle of the dorsal abdomen and hindwing of *T. castaneum* adults, characterized their genes and expression profiles, and analyzed their roles in adult cuticle formation and stabilization. We have also determined that these two CPR proteins are essential structural components in the sclerotized dorsal cuticle of the elytron and are required for normal morphological, functional and mechanical properties.

## Results

### Elytra from *T. castaneum* contain two predominant cuticular proteins

**Figure 1:**
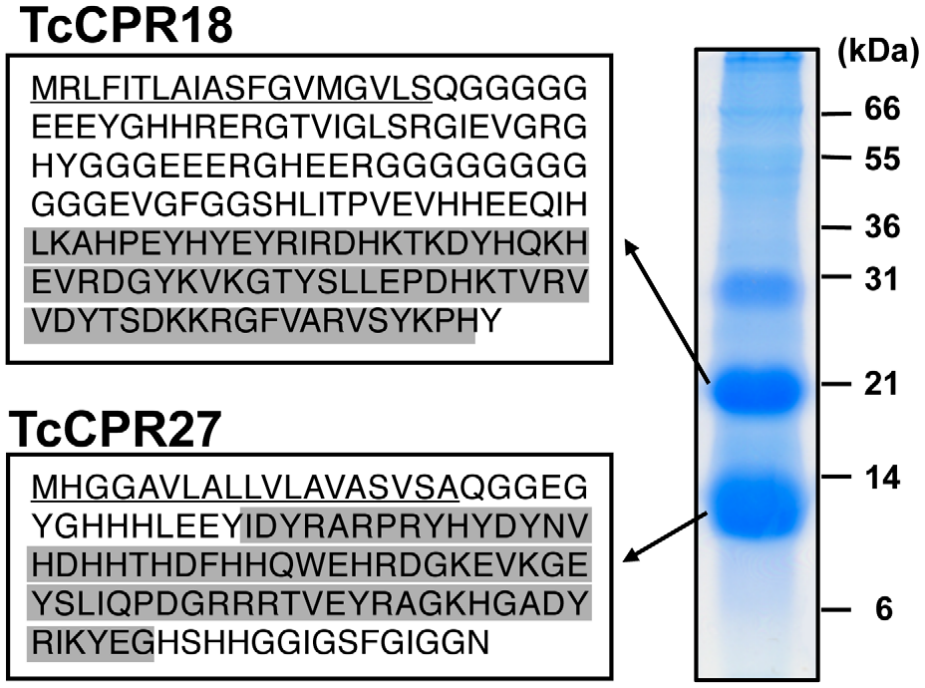
Identification of two major elytral cuticle proteins from *T. castaneum*. Extracts of SDS-soluble proteins from elytra dissected from newly emerged adults were analyzed by SDS-PAGE. The two major proteins (subsequently named TcCPR27 and TcCPR18), with apparent sizes of 10 and 20 kDa, respectively, were digested with trypsin, and the resulting peptides were analyzed by MALDI-TOF mass spectrometry (see Figure S1 and Table S1). Amino acid sequences deduced from cloned cDNA sequences for each protein are shown on the left. Both proteins contain an RR-2 motif (highlighted in gray). Predicted secretion signal peptides are underlined. In both TcCPR27 and TcCPR18, the amino-terminal residue of the mature forms after secretion (Gln 20) is apparently modified to pyroglutamic acid (Table S1).

Extracts of untanned elytra dissected from newly emerged adults contained two highly abundant proteins with apparent sizes of 10 and 20 kDa based on their electrophoretic mobilities (Figure 1). To characterize these major proteins further, each was digested with trypsin, and the resulting peptides were analyzed by MALDI-TOF/TOF mass spectrometry. Comparison of these results with conceptual trypsinization of the computed proteome of *T. castaneum* revealed two candidate genes, denoted as *TcCPR27* (XP_971678) and *TcCPR18* (XP_967633), which are members of the Rebers and Riddiford family of cuticular proteins (Figure 1, Table S1 and cuticle DB: http://biophysics.biol.uoa.gr/cuticleDB). Peptide coverages for TcCPR27 and TcCPR18 were 68.2 and 88.7%, respectively (Figure S1).

We cloned cDNAs corresponding to these cuticular protein genes (accession numbers HQ634478 and HQ634479). The cDNA sequence of *TcCPR27* was identical to that of the NCBI RefSeq gene prediction, whereas the RefSeq prediction for *TcCPR18* had one in-frame deletion of three consecutive nucleotides and a single nucleotide mismatch compared to the cDNA, resulting in a deletion of one amino acid (one of the twelve consecutive glycines at amino acid positions 65–76 in the RefSeq prediction) and a phenylalanine-to-leucine substitution at amino acid position 85 in the RefSeq prediction. *TcCPR27* and *TcCPR18* encode proteins containing putative secretion signal peptide sequences, with theoretical molecular masses for the mature proteins of 11.4 and 16.4 kDa, respectively.

Each mature protein contains a single RR-2 cuticular protein motif. Nearly all RR-2 proteins have a consensus region as follows: G-X(8)-G-X(6)-Y-X(6)-GF [7]. Both of these *Tribolium* proteins, however, have a slightly different RR2 motif. TcCPR27 contains G-X(8)-G-X(6)-Y-X(5)-GA, whereas TcCPR18 has G-X(8)-H-X(7)-Y-X(6)-GF. The former has a GA rather than the almost universal GF or GY at the end of the consensus, and the conserved Y and the third G are interrupted by five amino acids instead of the typical six. In the case of TcCPR18, the second conserved G is replaced by an H with no G residue located nearby.

TcCPR18 is rich in glycine (21.6%), whereas TcCPR27 has a high content of both glycine (16.5%) and histidine (15.5%). TcCPR18 is an apparent ortholog of the ecdysteroid-regulated, adult-specific cuticle protein acp22 of *Tenebrio molitor* (yellow mealworm), with 67% sequence identity (Figure S2) [20]. Both *TcCPR27* and *TcCPR18* map to linkage group 3 of the *T. castaneum* genome, but they are not tightly linked (BeetleBase: http://beetlebase.org) [21]. Elytra of three other *Tribolium* species, *T. brevicornis*, *T. confusum* and *T. freemani*, also contain predominant cuticular proteins with high amino acid sequence similarities to TcCPR27 and TcCPR18 (Figure S3 and Table S2).

### TcCPR27 and TcCPR18 are abundantly expressed in rigid cuticle but not in flexible cuticle

**Figure 2:**
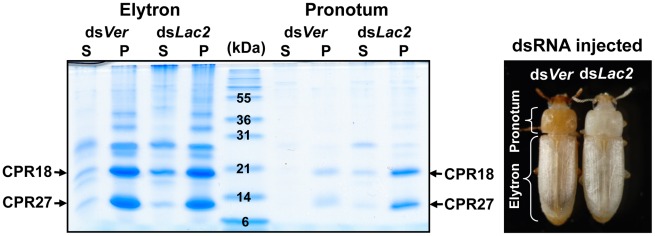
TcCPR27 and TcCPR18 are abundant in cuticle of the pronotum. To suppress cuticle tanning, dsRNA for *TcLac2* (2 ng per insect) was injected into day 0 pupae. Proteins from the pronotum and elytra were extracted from five adults at 0–30 min after eclosion, and the extracts were analyzed by SDS-PAGE (left panel). In the ds*Ver*-treated control animals, the extent of tanning of the pronotum was substantially greater than that of the elytron (right panel). S: PBS homogenate supernatant, P: PBS homogenate pellet.

To investigate whether TcCPR27 and TcCPR18 are present in other regions of the adult cuticle, we extracted proteins from cuticular samples dissected from the pronotum and the ventral abdomen just after adult eclosion. As in the case of the elytra, TcCPR27 and TcCPR18 proteins were also the predominant protein constituents of the pronotum, although their yields were low relative to those obtained from the elytra (Figure 2). We hypothesized that because the extent of tanning of the pronotum just after eclosion is substantially greater than that of the elytron, which tans at a later time (Figure 2), pronotum cuticular proteins were already cross-linked and much less extractable at the time of adult eclosion. To delay pronotum cuticle tanning, dsRNA for laccase-2 (ds*TcLac2*) was injected into 0–1 d-old pupae [2], and the pronotum cuticular proteins were subsequently extracted from samples obtained soon after adult eclosion. The yields of TcCPR27 and TcCPR18 were much higher in those extracts, indicating that the two proteins had not undergone substantial cross-linking in the absence of laccase and therefore were more readily extractable (Figure 2).

Like the elytron, the adult ventral abdominal cuticle undergoes tanning and becomes hardened 3–5 d post eclosion, whereas the dorsal abdominal cuticle in the adult remains relatively untanned, flexible and transparent like the hindwing. TcCPR27 and TcCPR18 were abundant in extracts recovered from ventral abdominal cuticle of newly emerged adults, but very little or no TcCPR27 or TcCPR18 was present in extracts of the dorsal abdominal cuticle (Figure S4A). Similarly, the levels of TcCPR27 and TcCPR18 in the flexible hindwing were very low or undetectable (Figure S4A). These results show that TcCPR27 and TcCPR18 are major proteins in cuticles that become highly sclerotized and rigid, but they are absent or only very minor components of cuticles that are more flexible and membranous.

### *TcCPR27* and *TcCPR18* genes are expressed at high levels in body regions with rigid adult cuticle

**Figure 3:**
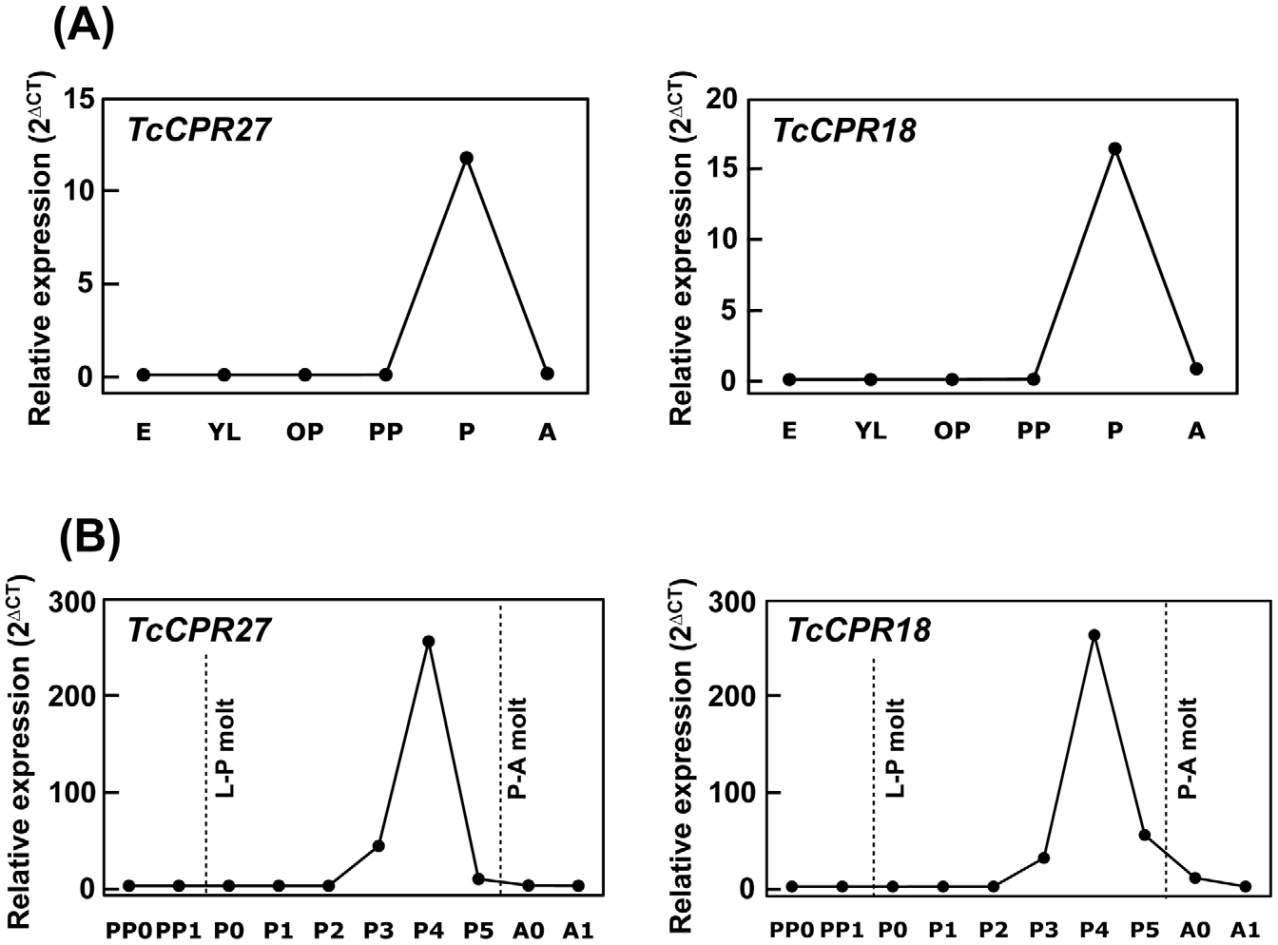
Expression profiles of *TcCPR27* and *TcCPR18* genes during development. (A) To analyze the expression profiles of *TcCPR27* and *TcCPR18* during development, real-time PCR experiments were done using total RNA extracted from five whole insects at different developmental stages (embryo to adult). Both genes were highly expressed at the pupal stage. (B) To analyze more rigorously the expression patterns of these genes, the stages analyzed were expanded between the early pharate pupal and young adult stages. The transcript levels of both genes dramatically increased at the pharate adult stage and declined rapidly thereafter. E, embryos; YL, young larvae; ML, mature larvae; PP, pharate pupae; P, pupae; A, adults; PP0, day 0–1 pharate pupae; PP1, day 1–2 pharate pupae; P0, day 0 pupae; P1, day 1 pupae, P2, day 2 pupae; P3, day 3 pupae; P4, day 4 pupae (pharate adults); P5, day 5 pupae (pharate adults); A0, day 0 adults; A1, day 7 adults. Expression levels for *TcCPR27* and *TcCPR18* are presented relative to the levels of expression in embryos (E) or 0–1 d old pharate pupae (PP0). The transcript levels of the *T. castaneum* ribosomal protein S6 (*rpS6*) were measured to normalize for differences between samples in the concentrations of cDNA templates.

Few or no transcripts for *TcCPR27* or *TcCPR18* were detected during the egg, larval or early pupal stages of development. However, the transcript levels of these genes dramatically increased at the pharate adult stage 0–1 d before eclosion, declining soon thereafter (Figure 3A, 3B). Transcript levels of *TcCPR27* and *TcCPR18* in the elytron were approximately 1,700- and 55-fold higher, respectively, than those in the membranous hindwing (Figure S4B). Both genes were also expressed in the pronotum and ventral abdomen, whose cuticles become highly sclerotized and hardened in mature adults. Transcript levels of *TcCPR27* and *TcCPR18* in the ventral abdomen were approximately 3,000 and 770 times higher, respectively, than the levels in the transparent, flexible and membranous dorsal abdomen (Figure S4B).

### TcCPR27 is localized in rigid cuticle of the dorsal elytra and ventral abdomen

**Figure 4:**
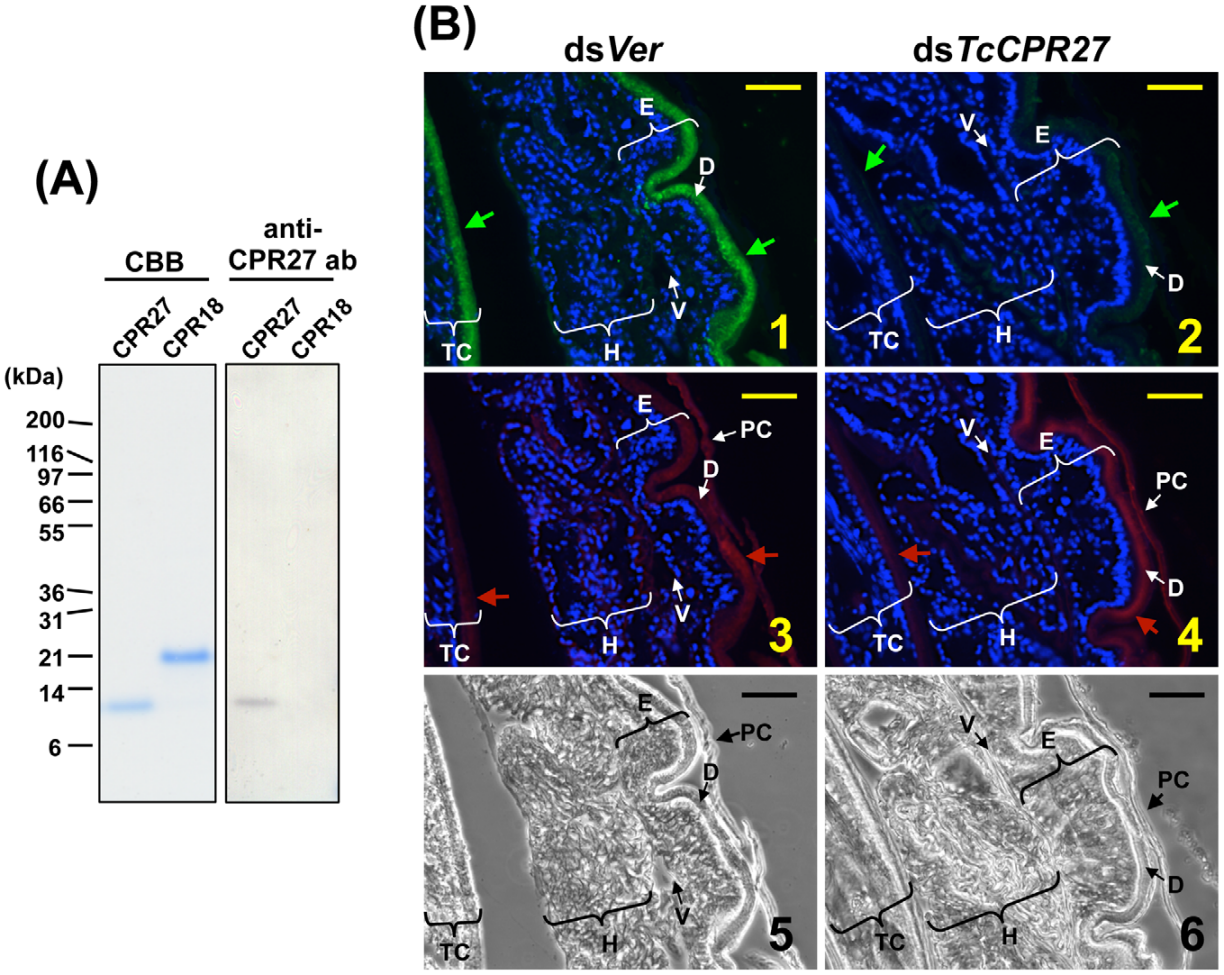
Immunolocalization of TcCPR27 in elytral cuticle. (A) Coomassie staining and immunoblot analyses of purified TcCPR27 and TcCRP18. (B) Immunolocalization of TcCPR27 in pharate adults. Cryosections (5–10 µm) of 5 d old pupae that had been injected previously with dsRNA for *TcCPR27* or *TcVer* (*T. castaneum Vermilion*) in the last larval instar were incubated with the anti-TcCPR27 antibody. Anti-TcCPR27 antibody was detected by Alexa Fluor 488-conjugated anti-rabbit IgG antibody (green arrows in panels 1 and 2). The same sections were also stained with a rhodamine-conjugated chitin-binding probe (red arrows in panels 3 and 4) [41]. Nuclei were stained with DAPI (blue). E = elytron, H = hindwing, TC = thoracic cuticle, PC = pupal cuticle, D = elytral dorsal cuticle, V = elytral ventral cuticle. Scale bar = 10 µm.

The high histidine content of TcCPR27 and TcCPR18 (15.5% and 10.1%, respectively) allowed us to purify these proteins from elytra dissected from newly molted adults by utilizing nickel-affinity chromatography (Figure 4A). A polyclonal antibody directed against purified TcCPR27 was then generated. The CPR27 antibody specifically detected CPR27 but not CPR18 (Figure 4A). In pharate adults, TcCRP27 was co-localized with chitin in the dorsal elytral cuticle as well as in the ventral abdominal cuticle, both of which become more rigid and darker as the adult matures (Figure 4B, panels 1, 3). Little or no TcCRP27 immunoreactivity was detected in the pupal, hindwing, or ventral elytral cuticles.

### RNAi–mediated knockdown of *TcCPR27* and *TcCPR18* expression leads to malformed and weakened elytra

**Figure 5:**
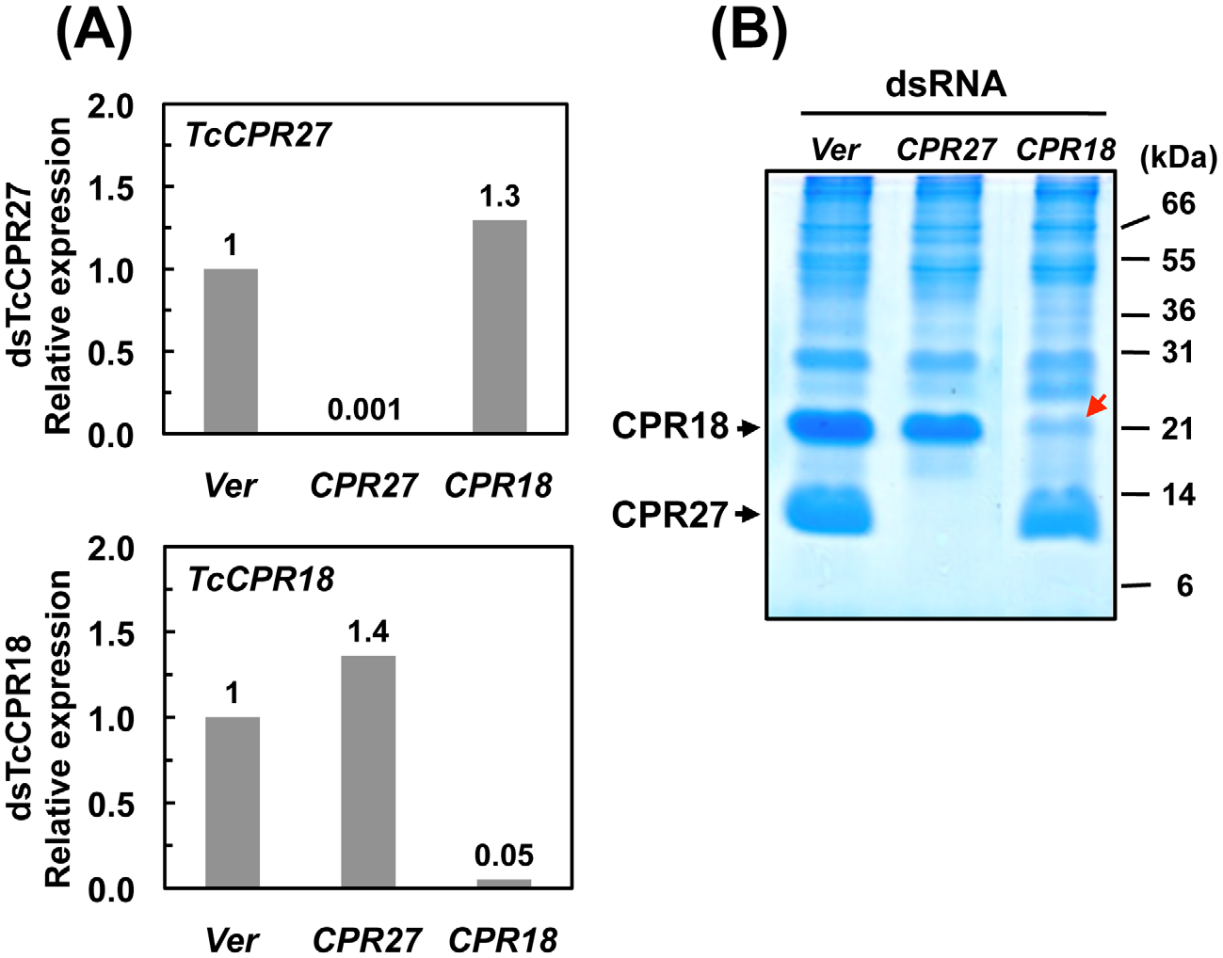
Knockdown of *TcCPR27* and *TcCPR18* transcripts by RNAi. Late instar *T. castaneum* larvae were injected with 0.2 µg of dsRNA for *TcCPR27* or *TcCPR18*. Following dsRNA injections, expression of *TcCPR27* and *TcCPR18* genes was analyzed by real-time PCR (A) and SDS-PAGE (B) to evaluate transcript and protein levels. cDNAs were prepared from total RNA isolated from five whole insects at pupal day 5 (10 d post-injection). For real-time PCR, expression levels of *TcCPR27* and *TcCPR18* are presented relative to the levels in *Ver* control insects (injected with dsRNA for *T. castaneum Vermilion gene*). The transcript levels of the *T. castaneum* ribosomal protein S6 (*rpS6*) were measured to normalize for differences between samples in the concentrations of cDNA templates. Proteins were extracted from elytra from five newly emerged adults for each treatment. A faint band (red arrow) with a mobility similar to that of TcCPR18 observed in extracts of whole insects injected with dsRNA for *TcCPR18* was identified by mapping of tryptic peptides by MALDI-TOF to be a different CPR RR2 protein, TcCPR33, with a theoretical mass of 19.1 kDa. These data indicate that both *TcCPR27*, *TcCPR18* were specifically down-regulated at both the mRNA and protein levels after dsRNA injections.

RNA interference (RNAi) was used to investigate the functions of *TcCPR27* and *TcCPR18*. As a negative control we injected dsRNA for *T. castaneum* tryptophan oxygenase (the *vermilion* gene, abbreviated *Ver*), a gene required for normal eye pigmentation [22]. Following dsRNA injections into last instar larvae, mRNA and protein levels of *TcCPR27* and *TcCPR18* were analyzed by real-time PCR and SDS-PAGE. Injection of these dsRNAs led to substantial and specific down-regulation of each gene at the mRNA (Figure 5A) and protein (Figure 5B) levels. TcCPR27 immunostaining also was strongly reduced after injection of ds*TcCPR27* (Figure 4B, panel 2). Chitin staining in TcCPR27-deficient insects, however, was detected at approximately the same level as in ds*Ver*-treated control animals (Figure 4B, panel 4). These results were further supported by staining of elytral chitin with a fluorescent chitin-labeling reagent, FITC-CBD. There was no difference in chitin staining among elytra collected from ds*TcCPR27*-, ds*TcCPR18*- and ds*Ver*-treated insects (Figure S5).

**Figure 6:**
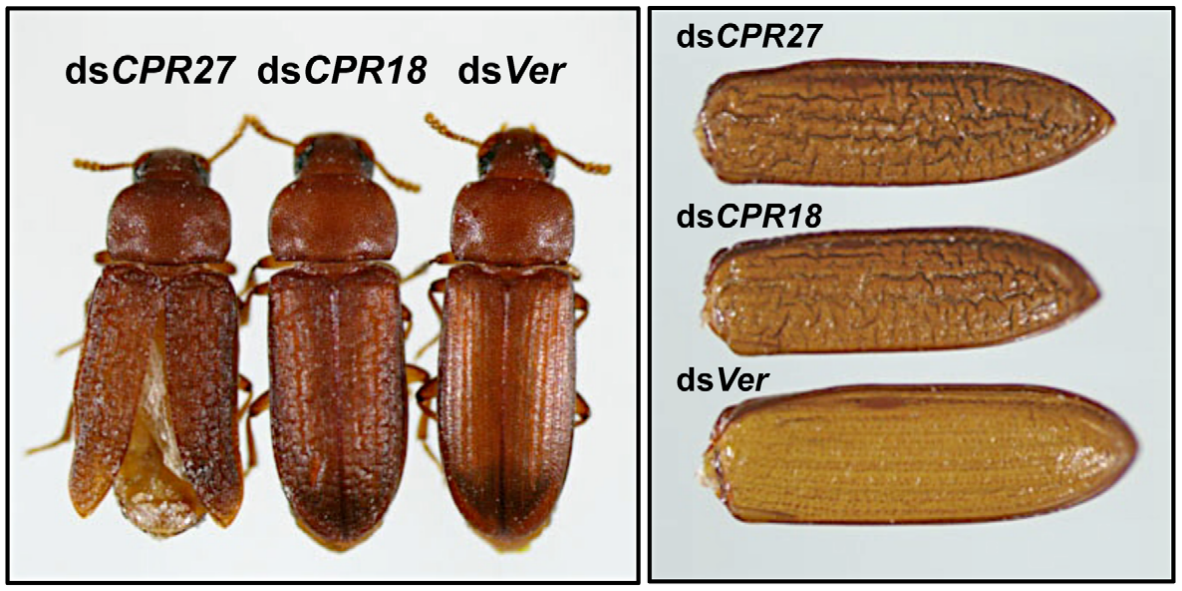
*T. castaneum* elytral defects produced by injection of dsRNAs for *TcCPR27* and *TcCPR18*. To investigate the functions of *TcCPR27* and *TcCPR18*, specific dsRNAs for *TcCPR27* (ds*CPR27*) or *TcCPR18* (ds*CPR18*) (0.2 µg per insect) were injected into late instar larvae. Dorsal views of the resulting adults (1-d old) (left panel) and elytra (right panel) are shown. dsRNA for *Ver* (ds*Ver*) was injected to serve as a negative control.

Injection of ds*TcCPR27* or ds*TcCPR18* into larvae had no apparent effect on larval-pupal or pupal-adult molting or on the morphology of the pupal cuticle, as expected from the observed late pupa-specific expression of these genes. However, the elytra of the resulting adults were malformed (Figure 6 and Figure S6). The surface of the elytra of ds*TcCPR18*-treated adults was irregular and rough compared to those of control insects. Adults derived from ds*TcCPR27*-injected larvae exhibited even more severe morphological defects. Their elytra were very short, wrinkled, warped and fenestrated (Figure 6 and Figure S6) and their hindwings were unable to fold normally. Such insects died approximately one week after eclosion, apparently from dehydration that resulted from failure of the misshapen elytra to properly cover the membranous dorsal abdomen and to thereby seal it against trans-cuticular water loss. The shape of a normal elytron is “inverted boat-like” to fit snugly on top of the hindwings and abdomen in order to protect the latter structures (Figure S6). In contrast, elytra from ds*TcCPR27*-treated insects were flatter and/or warped and did not cover the entire abdomen. Manual excision of the distal half of the elytron from a mature adult also led to high mortality, whereas removing an entire hindwing did not cause significant mortality, as long as the elytra could adopt their normal juxtaposition (Figure S7), consistent with our observation that properly formed elytra are essential to prevent desiccation of the adult, in addition to other potentially protective functions. These results support the hypothesis that TcCPR27 and TcCPR18 are major structural proteins in rigid elytral cuticle, and are required for normal elytral morphogenesis, hindwing folding and body hydration.

### Effect of dsRNA for TcCPR27 and TcCPR18 on the mechanical properties of elytra

**Figure 7:**
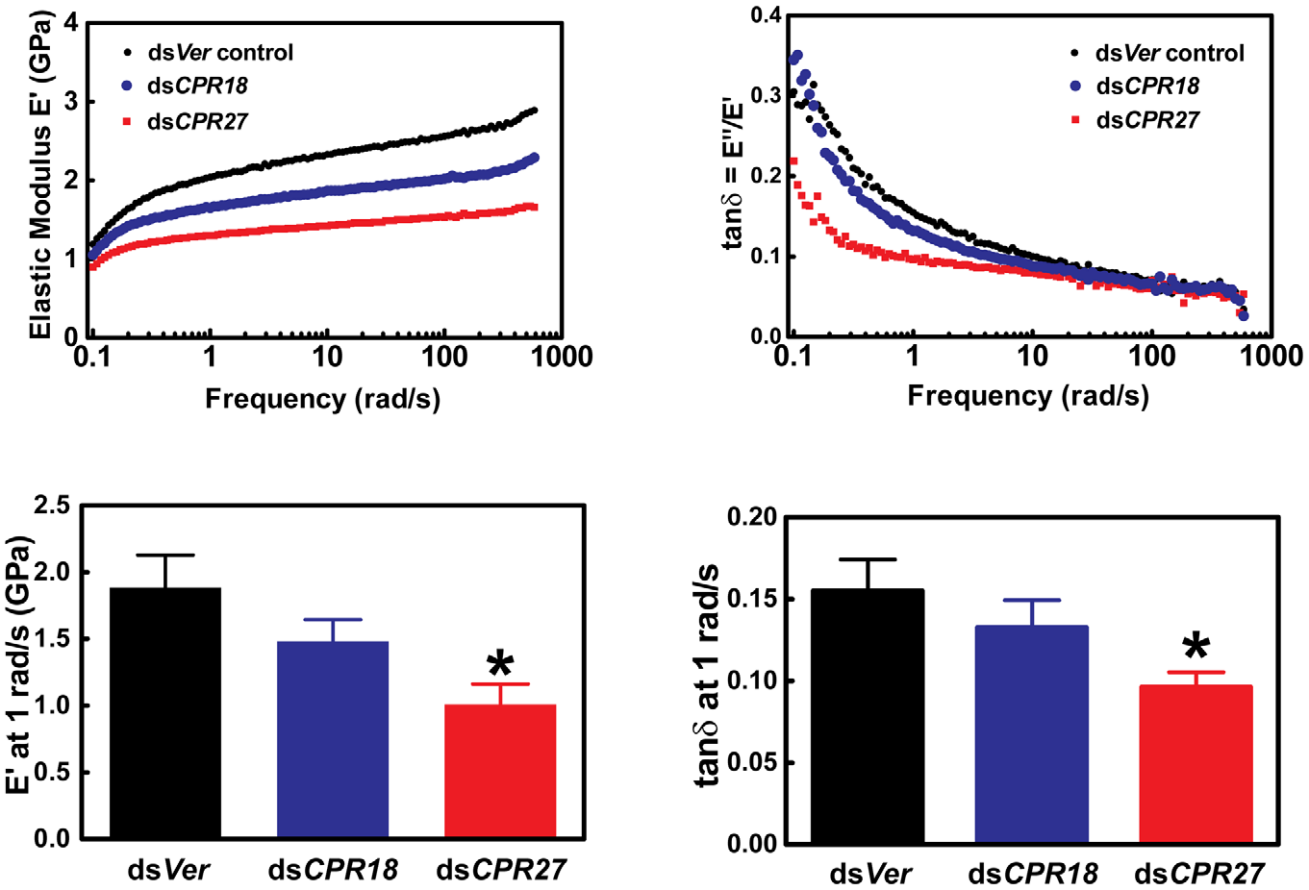
Mechanical properties of *Tribolium* ds*TcCPR27* and ds*TcCPR18* elytra. Elytra from beetles on day 2 after adult eclosion were examined by dynamic mechanical analysis over a frequency range of 0.1 to 600 rad/s. Typical scans are shown in the top panels, and mean values at 1 rad/s are presented in the lower panels. Error bars represent standard deviation (n = 3–4). The asterisk indicates significant difference from the ds*Ver* control (p<0.05) as determined by analysis of variance and Tukey's Multiple Comparison Test. Reduced expression of the abundant CPR proteins resulted in weaker (smaller E′) yet more cross-linked (smaller tan δ) elytra.

We also analyzed the effects of depletion of TcCPR27 and TcCPR18 on the mechanical properties of elytra. Dynamic mechanical experiments were carried out to determine the storage modulus E′ and the loss modulus E″ of elytra as a function of oscillation frequency and strain. E′ is a measure of the elastically recoverable deformation energy, whereas E″ is a measure of viscous energy dissipation (dampening) and hence is also known as the viscous modulus. The ratio E″/E′ is known as the “loss tangent” or simply tan δ, where δ is the phase angle between sinusoidally applied stress and strain. For materials such as the elytra where E′>>E″, E′ is approximately equal to the Young's modulus obtained from the slope of simple stress-strain measurements at the same strain rate [23], [24]. Hence, E′ is a measure of the stiffness of the elytra. Elytra from animals injected with ds*TcCPR27* were significantly less rigid (lower E′) than the ds*Ver*-treated controls (Figure 7). Elytra from ds*TcCPR18*-injected beetles had intermediate values of strength, consistent with the less severe visible phenotype observed with *TcCPR18* knockdown (Figure 6). In addition, the ds*TcCPR27* elytra had reduced values for tan δ, an indication that they experienced a higher degree of cross-linking than the control. Lower E″ or tan δ in polymeric networks is typically associated with a reduction in the network of uncross-linked material, dangling chains (chains linked to the network at only one end), loops and other network imperfections [25]. It is a well-established principle in the synthesis of gels or networks by cross-linking polymers, that increasing the ratio of cross-linker molecules to polymer molecules will typically increase the overall cross-link density of a network and reduce the fraction that is not cross-linked [26]. Thus, for elytral cuticle, the deficiency of a major structural cuticular protein such as TcCPR27, while maintaining a constant concentration of quinone cross-linking molecules, would be expected to lead to a greater average number of cross-links per protein molecule. Thus, for elytral cuticle, reducing the expression level of a major structural cuticular protein such as TcCPR27 or TcCPR18, while maintaining a constant concentration of quinone cross-linking molecules, would be expected to lead to a greater average number of cross-links per protein molecule. The more severe phenotype for knockdown of TcCPR27, relative to TcCPR18 might be due to differences in the degree of knockdown of their expression in the RNAi experiments, or perhaps could be due to differences in their structural properties or cross-linking chemistry. A greater reduction in protein levels for TcCPR27 than TcCPR18 in the knockdown animals might have led to relatively more cross-linking, thus reducing tan δ, and a greater reduction in TcCPR27 protein expression would lead to a lower storage modulus. Similar observations were previously reported for elytra from insects subjected to *Lac2* knockdown (reduced tan δ combined with reduced E′) [19].

## Discussion

The basic genetic patterning mechanism for dorsal appendages such as wings has been described for both *D. melanogaster* and *T. castaneum* [27]–[29]. Similar gene networks are used to pattern wings, elytra and halteres, despite the profound morphological and functional divergence of these appendages during insect evolution. Individual structural proteins are likely to substantially affect the physical properties of elytra. However, to date, there have been no detailed reports about the contributions of individual structural proteins to elytral morphogenesis.

Like other beetle species, *T. castaneum* adults possess elytra, modified forewings with a highly sclerotized and pigmented dorsal cuticle. Immediately after eclosion, untanned elytra have a soft white cuticle. Elytra expand shortly thereafter and then become rigid and darker during cuticle maturation. The role of structural proteins in this developmental process is the focus of this study. We identified two proteins from the RR2 family of cuticular proteins, TcCPR27 and TcCPR18, which are highly abundant in protein extracts of elytra dissected from newly emerged adults. TcCPR27 and TcCPR18 transcripts were strongly up-regulated at the developmental stage when adult cuticular proteins are expected to be synthesized (pupal day 4), and were nearly absent at earlier immature stages. Highly abundant cuticular proteins related to TcCPR27 and TcCPR18 were also present in protein extracts of elytra dissected from three other *Tribolium* species including *T. brevicornis*, *T. confusum* and *T. freemani* (Figure S3 and Table S2).

TcCPR27 and TcCPR18 were also identified in extracts of pronotum cuticle, in which tanning had been initiated before adult eclosion. The protein yields, however, were much lower than those obtained from elytral extracts unless cuticle tanning was suppressed by injection of dsRNA for the tanning enzyme TcLac2 (Figure 2). These results support the hypothesis that TcCPR27 and TcCPR18 are cross-linked by highly reactive quinones and quinone methides that are produced by the cuticle tanning phenoloxidase laccase-2, and that these proteins become inextractable after tanning has occurred. Previously, Missios *et al.* [30] extracted two major cuticular proteins of 10 and 20 kDa, consistent with the apparent masses of TcCPR27 and TcCPR18, respectively, from extracts of cuticle from whole bodies of newly eclosed *T. castaneum* adults. Neither of these proteins was extractable from 7 day-old adults, consistent with an interpretation that these proteins become cross-linked during maturation of the cuticle.

To study the functions of *TcCPR27* and *TcCPR18*, we performed RNAi and successfully down-regulated levels of *TcCPR27* and *TcCPR18* mRNAs and proteins (Figure 5). These deficiencies caused several elytral defects. Although TcCPR27 and TcCPR18 are also present in other body regions such as the cuticles of the pronotum and ventral abdomen, which are heavily tanned in the mature adult, we did not observe visible phenotypic changes in those cuticles after injection of ds*TcCPR27* and ds*TcCPR18*. The size and shape of these body regions do not change much after adult eclosion, in contrast to the elytra that are greatly expanded shortly after eclosion. The elytra of both TcCPR27- and TcCPR18-deficient insects failed to fully expand, and their dorsal surfaces were not smooth (Figure S6). The elytra of TcCPR27-deficient insects, particularly, were very short, wrinkled and fragile. Elytra from ds*TcCPR2*7- and ds*TcCPR18*- treated insects appear to contain more cross-linked proteins than the elytra from ds*Ver*-treated control insects (Figure 6). Lacking these major cuticular proteins apparently increases the effective concentration of cross-linking agents (NADA and NBAD quinones), resulting in aberrant cross-linking among the remaining proteins and shortened warped elytra. The effect was seen most clearly in ds*TcCPR2*7-treated insects, in which the modulus and tan δ were significantly reduced relative to control insects. The ds*TcCPR18*- treated insects had a smaller decrease in modulus and tan δ, perhaps because of a smaller degree of protein reduction. The difference could also arise from structural differences between TcCPR27 and TcCPR18, which could have different propensities for forming intermolecular vs. intramolecular cross-links. However, the present data cannot draw that level of distinction. All of these results suggest that TcCPR27 and TcCPR18 are critical for normal elytral morphogenesis and are required to prevent dehydration and death of the adult.

In summary, we have identified in beetles two major structural proteins, TcCPR27 and TcCPR18, which account for approximately half of the extractable cuticular proteins in the elytra and also are major components of other hard cuticular structures. It is interesting to note that the proteins utilized for hard cuticles of other body regions of the beetle were apparently used to build the elytron's hard cuticle. In some saturniid moth species, proteins from the same CPR family are also used to form rigid structures such as tubercles, head capsules and hard pupal cuticle [31]. We now have biomechanical evidence on just how important these kinds of proteins are.

TcCPR27 and TcCPR18 are required not only for rigid cuticle development, but also for morphogenesis, elytral mechanical properties, and survival of the red flour beetle. In contrast, these proteins are essentially undetectable in soft cuticles. Expression of such cuticular proteins in the modified forewings appears to be a fundamental evolutionary step in transforming the flexible and thin membranous wing into a thickened and rigid elytron in the Coleoptera. In the case of *TcCPR18*, an orthologous gene is found in the only other beetle species examined, the lesser grain borer, *Rhyzopertha dominca*, in the family Bostrichidae (Schlipalius, D. and Beeman, R. W., unpublished observations) but not in any of the other sequenced arthropod genomes, including representatives of the Diptera, Hymenoptera and Lepidoptera. These structural proteins are probably cross-linked during sclerotization, via formation of histidyl-catechol adducts [32], [33]. Rigidification of the beetle forewing has likely been achieved in part through both structural protein incorporation and multiple co-options of the sclerotization pathway acting downstream of conserved wing gene network components, with the final product being primarily a rigid interpenetrating network of chitin embedded in a cross-linked protein matrix [3], [29], [34], [35]. To gain a more comprehensive understanding of the roles of cuticular proteins in defining the morphology and properties of the beetle elytron and rigid body wall cuticle, future studies are required to determine, at the ultrastructural level, the precise localization of TcCPR27, TcCPR18 and other structural proteins, and to assess the nature and extent of their covalent cross-linking during sclerotization.

## Materials and Methods

### Insects

The GA-1 strain of *T. castaneum* was used in this study. Beetles were reared at 30°C under standard conditions [36].

### Protein extraction and identification

Elytra of newly emerged adults (n = 10) were homogenized in 100 µl of cold PBS containing protease inhibitor cocktail (Roche). The homogenate was centrifuged for 2 min at 4°C. The supernatant was collected as PBS soluble fraction. The pellet was homogenized in 100 µl of SDS-PAGE sample buffer, heated at 95°C for 10 min, centrifuged for 2 min. The supernatant was collected as PBS pellet fraction. Protein extracts were analyzed by 15% SDS-PAGE or 4–12% Bis-Tris gradient gel (Invitrogen). Proteins were digested with trypsin, and the resulting fragments were analyzed by MALDI-TOF mass spectrometry.

### Identification of proteins by mass spectrometry

After staining gels with Coomassie G-250, the selected gel band was excised as 1–2 mm diameter pieces and transferred to a 1.5 mL Eppendorf tube. A protein-free region of the gel was also excised as background control. The control and test gel sections were destained using three 30 min washes of 60 µL 1∶1 acetonitrile: water at 30°C. Gel pieces were then dried for 10 min under vacuum. The gel sections were subjected to reduction and alkylation using 50 mM Tris (2-carboxyethyl) phosphine (TCEP) at 55°C for 10 min followed by 100 mM iodoacetamide in the dark at 30°C for 60 min. The carboxymethylated gels were thoroughly washed and re-dried *in vacuo*, then incubated with sequencing grade trypsin (Trypsin Gold, Promega, Madison, WI), 20 ng/µL in 40 mM ammonium bicarbonate, in 20 µL. Upon rehydration of the gels, an additional 15 µL of 40 mM ammonium bicarbonate and 10% acetonitrile was added, and gel sections were incubated at 30°C for 17 h in sealed Eppendorf tubes. The aqueous digestion solutions were transferred to clean 1.5 mL Eppendorf tubes, and tryptic fragments remaining within the gel sections were recovered by a single extraction with 50 µl of 50% acetonitrile and 2% trifluoracetic acid (TFA) at 30°C for 1 h. The acetonitrile fractions were combined with previous aqueous fractions and the liquid was removed by speed vacuum concentration. The dried samples were resuspended in 10 µL of 30 mg/mL 2,5-dihydroxylbenzonic acid (DHB) (Sigma, St. Louis, MO) dissolved in 33% acetonitrile/0.1% TFA and 2 µL of peptide/matrix solution was applied on a Bruker Massive Aluminum plate for MALDI-TOF and TOF/TOF analysis. Mass spectra and tandem mass spectra were obtained on a Bruker Ultraflex II TOF/TOF mass spectrometer. Positively charged ions were analyzed in the reflector mode. MS and MS/MS spectra were analyzed with Flex analysis 3.0 and Bio Tools 3.0 software (Bruker Daltonics). Measurements were externally calibrated with 8 different peptides ranging from 757.39 to 3147.47 (Peptide Calibration Standard I, Bruker Daltonics) and internally recalibrated with peptides from the autoproteolysis of trypsin. Peptide ion searches were performed with Beetlebase (http://www.bioinformatics.ksu.edu/BeetleBase/) (as well as Metazoa domain_201000104 in NCBInr database) using MASCOT software (Matrix Science). The following parameters were used for the database search: MS and MS/MS accuracies were set to <0.5 Da, trypsin/P as an enzyme, missed cleavages 1, carbamidomethylation of cysteine as fixed modification, and oxidation of methionine as a variable modification. Sequence motif analysis of the predicted protein sequence was searched in motifs database including PROSITE profiles and Pfam HMMs.

### *TcCPR27* and *TcCPR18* cDNAs

The full-length coding sequences for *TcCPR27* and *TcCPR18* (351 bp and 504 bp, respectively) were amplified from total RNA extracted from pupae (mixture of 0 d- to 5 d-old pupae) by RT-PCR. The cDNAs for *TcCPR27* and *TcCPR18* were amplified using the following gene specific primers, which included predicted start and stop codons: 5′ ATG CAC GGT GGA GCA GTT C 3′ and 5′ TCA GTT GCC TCC AAT CCC G 3′ for *TcCPR27*, and 5′ ATG AGA TTA TTT ATT ACA TTG GCC 3′ and 5′ CTA GAT TAA TAA TGT GGT TTG TAA G 3′ for *TcCRP18*. PCR products were cloned into pGEMT (Promega) and sequenced.

### Real-time PCR

Total RNA isolation, cDNA synthesis and real-time PCR were done as described previously [37] using the following primer sets: 5′AGG TTA CGG CCA TCA TCA CTT GGA 3′ and 5′ATT GGT GGT GGA AGT CAT GGG TGT 3′ for *TcCPR27*, 5′ GAA TAC CGC ATC CGT GAC CAC AAA 3′ and 5′CAG GTT CCA ACA AAC TGT AGG TTC CC 3′ for *TcCPR18*. Total RNA was isolated from whole insects (n = 5) to analyze developmental expression patterns and knock-down levels after RNAi of *TcCPR27* and *TcCPR18*. Total RNA also was isolated from elytra, hindwings, ventral abdomens and dorsal abdomens of pharate adults (5 d-old pupae) (n = 10). The transcript levels of the *T. castaneum* ribosomal protein S6 (*rpS6*) were measured to normalize for differences between samples in the concentrations of cDNA templates.

### Double-stranded RNA synthesis and injection

dsRNA for *TcCPR27* and *TcCPR18* was synthesized as described previously [38] using the primers 5′-(T7)-GAC CAC CAC ACC CAT G-3′ and 5′-(T7)-TCA GTT GCC TCC AAT C-3′ for *TcCPR27*, and 5′-(T7)-GGA AGA GTA CGG TCA TC -3′ and 5′-(T7)-GGT TCC CTT TAC TTT G-3′ for *TcCPR18*, where T7 indicates the T7 RNA polymerase recognition sequence. The sizes of dsRNAs for *TcCPR27* and *TcCPR18* were 204 bp and 325 bp, respectively. dsRNAs were injected into last instar larvae [39]. dsRNA for the *T. castaneum vermilion* gene (ds*Ver*) was used as a negative control [40].

### Purification of TcCPR27 and TcCPR18 from extracts of elytra

Proteins were extracted from 200 pairs of elytra of 5 d-old pupae as described in Materials and Methods. The homogenate was centrifuged for 2 min at 4°C. The supernatant was applied to a Ni-NTA column equilibrated with 50 mM Tris-HCl, pH 7.5 containing 0.2 M NaCl and 20 mM imidazole and washed with the same buffer. Bound proteins were eluted with a 20 to 200 mM imidazole gradient. The fractions were analyzed for protein content by SDS-PAGE. Purified TcCPR27 was used as antigen to generate rabbit antiserum by Cocalico Biologicals, Inc., PA, USA.

### Mechanical analysis of elytra

Mechanical analysis of elytra was carried out using a TA Instruments RSAIII dynamic mechanical analyzer, by methods described previously [34].

### Accession numbers

cDNA sequences are deposited at NCBI with accession numbers HQ634478 (TcCPR27) and HQ634479 (TcCPR18).

## Supporting Information

Figure S1 Trypsinization and peptide mass fingerprinting (PMF) by TOF-MS. Two major elytral cuticular proteins were digested with trypsin and the resulting peptides were analyzed by MALDI-TOF mass spectrometry. Results were compared with conceptual trypsinization products of the computed proteome of *T. castaneum*. Matched peptides are shown in red. Coverage for TcCPR27 and TcCPR18 was 88.7 and 68.2%, respectively. Underlined residues are predicted signal peptides, which are not included in the theoretical molecular mass calculations.
(TIF)

Figure S2 Amino acid sequence alignment of TcCPR18 and *Tenebrio molitor* adult-specific protein, Tmacp22. Alignment of deduced amino acid sequences was made using ClustalW software. The symbols below the aligned amino acid sequences indicate identical (*), highly conserved (:) and conserved (.) amino acids. TcCPR18 is a putative ortholog of the *T. molitor* ecdysteroid-regulated adult-specific cuticle protein, TmACP22, with 67% sequence identity and 74% similarity.
(TIF)

Figure S3 Highly abundant proteins similar to TcCRP27 and TcCPR18 are predominant cuticular proteins in elytra of other *Tribolium* species. Extracts of elytra from newly emerged adults of *T. castaneum*, *T. brevicornis*, *T. confusum* and *T. freemani* as well as *Tenebrio monitor* were analyzed by 4–12% Bis-Tris gel (Invitrogen). Like *T. castaneum*, two to three abundant proteins with the apparent masses of approximately 10 and 20 kDa were obtained from each species. These major proteins were digested with trypsin, and the resulting peptides were analyzed by MALDI-TOF mass spectrometry (see Table S2). The green and orange arrows indicate bands that exhibited high scores for similarity to TcCRT27 and TcCRT18, respectively. *T. monitor* adult cuticle proteins, TmACP20 and TmACP22 [20], were also identified. S: PBS homogenate supernatant, P: PBS homogenate pellet, M: protein size markers.
(TIF)

Figure S4 Expression patterns of *TcCPR27* and *TcCPR18* in the adult ventral vs. dorsal abdominal cuticles and elytra vs. hindwings. (A) The ventral and dorsal abdominal cuticles were dissected from five 0–30 min old adults. TcCRP27 and TcCRP18 were identified by peptide mapping in the ventral abdominal cuticle but not in the dorsal abdominal cuticle. S: PBS homogenate supernatant, P: PBS homogenate pellet. (B) To analyze the transcript levels of *TcCPR27* and *TcCPR18* in the ventral and dorsal abdomen as well as in the elytra and hindwings, real-time PCR was done using total RNA extracted from tissues of ten pharate adults (5 d-old pupae). Expression levels for *TcCPR27* and *TcCPR18* are presented relative to the levels of expression in elytra (E) or ventral abdomen (V). The transcript levels of the *T. castaneum* ribosomal protein S6 (*rpS6*) were measured to normalize for differences between samples in the concentrations of cDNA templates. H: hindwings, D: dorsal abdominal cuticle.
(TIF)

Figure S5 Elytral chitin staining with FITC-CBD. Elytra were removed from pharate adults (5 d-old pupae) that had been injected dsRNA for *TcCPR27*, *TcCPR18* or *TcVer* (200 ng per insect) at the late larval instar stage. The elytra were incubated with 10 N NaOH at 95°C for 5 h to remove protein, followed by staining with the fluorescein-conjugated chitin-binding domain probe (FITC-CBD, New England BioLabs) [38]. The appearance of the elytra did not differ until after adult eclosion, although ds*TcCPR27*- and ds*TcCPR18*-elytra were remarkably soft and fragile compared with ds*Ver*-elytra. The fluorescence was observed using a Leica MZ FLIII fluorescence stereomicroscope equipped with the following filter set: excitation = 480/40 nm, barrier = 510 nm.
(TIF)

Figure S6 Scanning electron micrographs of TcCPR27- and TcCPR18-deficient elytra. Elytra were dissected from 1 d-old adults that had been injected with dsRNA for *TcCPR27*, *TcCPR18* or *TcVer* (200 ng per insect) as last instar larvae. The dorsal view of elytra is shown. dsRNA for *Ver* was injected to serve as a negative control.
(TIF)

Figure S7 Survival rate after removing elytra or hindwings from mature *T. castaneum* adults. Elytra or hindwings were removed from mature adults (n = 20), and viability was monitored (insects were reared at 30°C and 50% humidity). A: whole hindwings removed. B: half of distal part of elytra removed. C: whole elytra removed. Loss of an entire hindwing did not affect adult survival, whereas removing elytra resulted in high mortality, probably because of dehydration. Thus, the elytra but not hindwings are essential for *T. castaneum* adult viability. Yellow and red lines indicate moribund and dead adults, respectively.
(TIF)

Table S1 Major proteins identified in extracts of unsclerotized elytra. Based on the cDNA sequence, the amino-terminal amino acid residue for both proteins is predicted to be a glutamine (Figure 1). The observed mass of native 10 kDa band was 11,467 Da as determined by MALDI-linear-TOF MS, suggesting that amino-terminus of mature protein of TcCPR27 might be a modified glutamine. Further analysis of MS and MS/MS profiles revealed that the most likely candidate for the amino-terminal residue is pyroglutamic acid. The tryptic peptide of 2143.1 Da from TcCPR27 corresponds to pyro-E_20_GGEGYGHHHLEEYIDYR_37_. A similar amino-terminal modification was confirmed in the 20 kDa band identified as TcCPR18 (1451.5 Da peptide, pyro-E_20_GGGGGEEEYGHHR_33_). These results suggest that for both proteins, Gln 20 is both deamidated and then dehydrated to form the observed modification.
(DOC)

Table S2 Identification of major proteins extracted from elytra of three *Tribolium* species.
(DOC)
