# Supplementary figures and images for: Formation of Rigid, Non-Flight Forewings (Elytra) of a Beetle Requires Two Major Cuticular Proteins
Source: PLoS Genet. 2012 Apr 26;8(4):e1002682. doi: 10.1371/journal.pgen.1002682 (PMC3343089; doi:10.1371/journal.pgen.1002682)

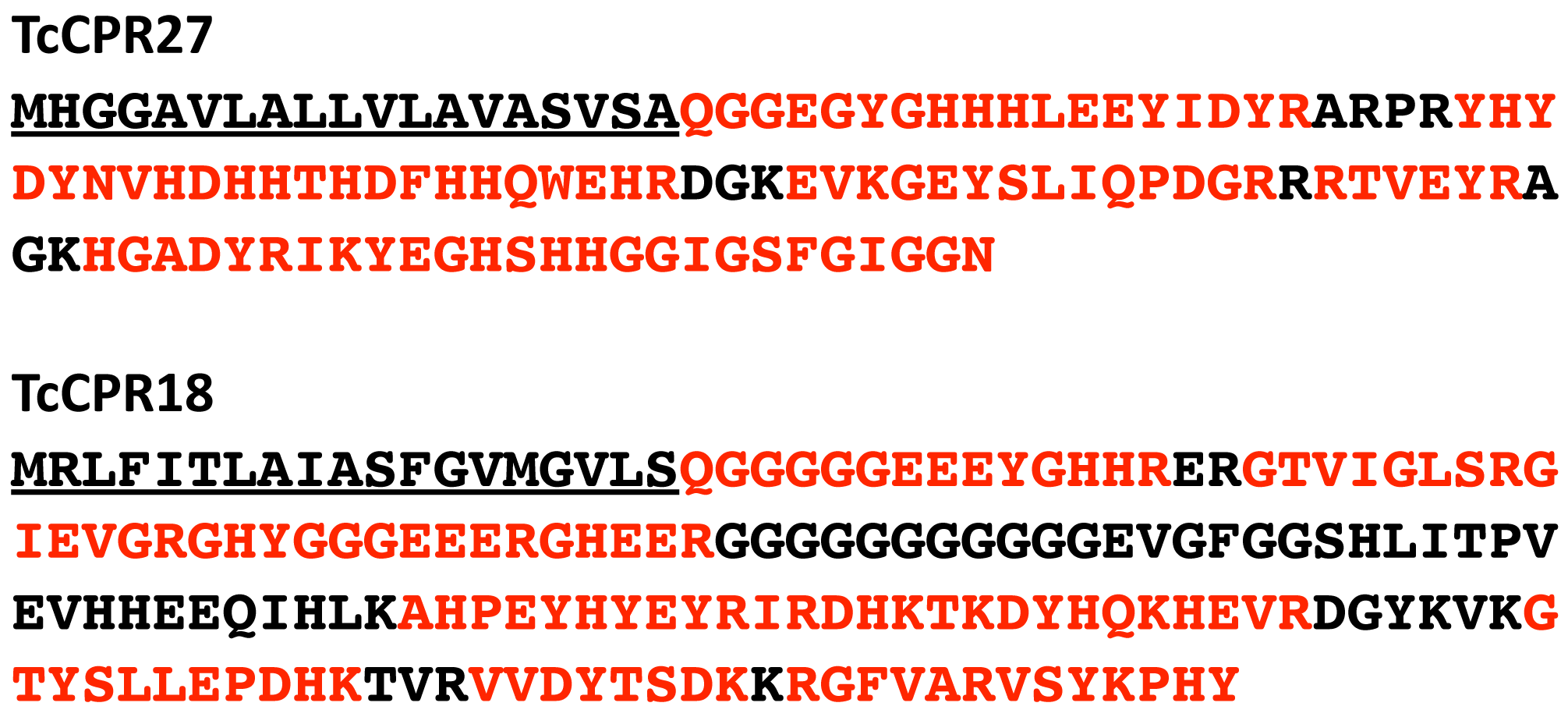

Supplement: Figure S1 — Trypsinization and peptide mass fingerprinting (PMF) by TOF-MS. Two major elytral cuticular proteins were digested with trypsin and the resulting peptides were analyzed by MALDI-TOF mass spectrometry. Results were compared with conceptual trypsinization products of the computed proteome of T. castaneum. Matched peptides are shown in red. Coverage for TcCPR27 and TcCPR18 was 88.7 and 68.2%, respectively. Underlined residues are predicted signal peptides, which are not included in the theoretical molecular mass calculations. (TIF) [file pgen.1002682.s001.tif]

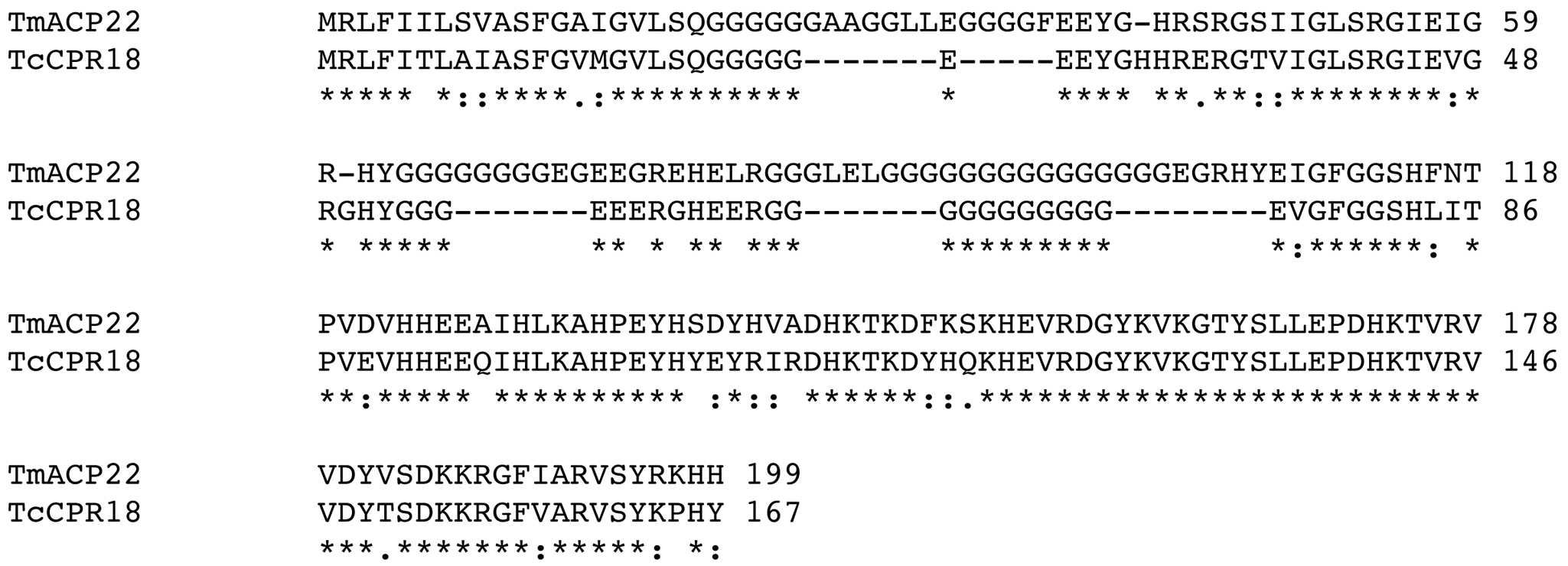

Supplement: Figure S2 — Amino acid sequence alignment of TcCPR18 and Tenebrio molitor adult-specific protein, Tmacp22. Alignment of deduced amino acid sequences was made using ClustalW software. The symbols below the aligned amino acid sequences indicate identical (*), highly conserved (:) and conserved (.) amino acids. TcCPR18 is a putative ortholog of the T. molitor ecdysteroid-regulated adult-specific cuticle protein, TmACP22, with 67% sequence identity and 74% similarity. (TIF) [file pgen.1002682.s002.tif]

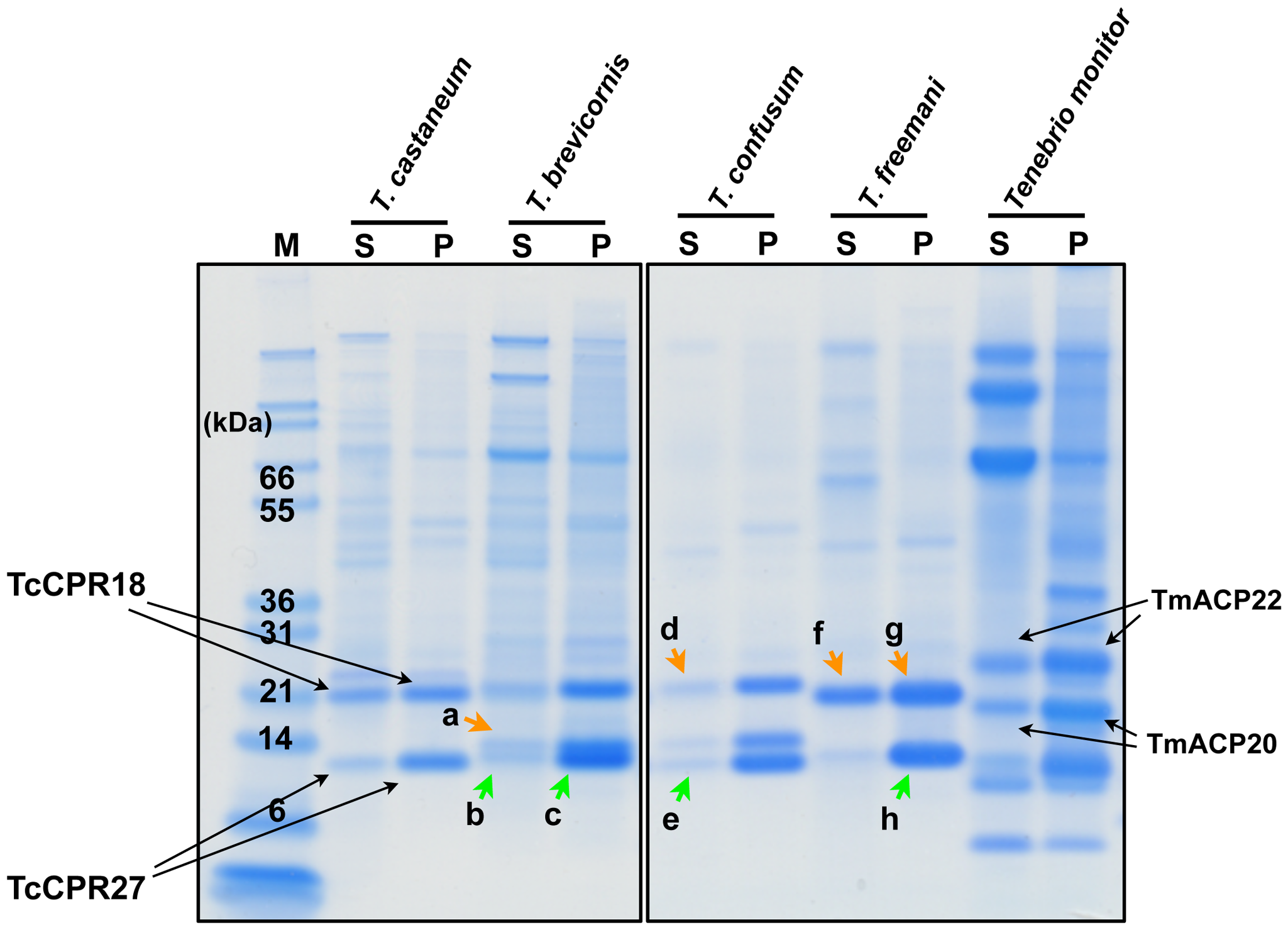

Supplement: Figure S3 — Highly abundant proteins similar to TcCRP27 and TcCPR18 are predominant cuticular proteins in elytra of other Tribolium species. Extracts of elytra from newly emerged adults of T. castaneum, T. brevicornis, T. confusum and T. freemani as well as Tenebrio monitor were analyzed by 4–12% Bis-Tris gel (Invitrogen). Like T. castaneum, two to three abundant proteins with the apparent masses of approximately 10 and 20 kDa were obtained from each species. These major proteins were digested with trypsin, and the resulting peptides were analyzed by MALDI-TOF mass spectrometry (see Table S2). The green and orange arrows indicate bands that exhibited high scores for similarity to TcCRT27 and TcCRT18, respectively. T. monitor adult cuticle proteins, TmACP20 and TmACP22 [20], were also identified. S: PBS homogenate supernatant, P: PBS homogenate pellet, M: protein size markers. (TIF) [file pgen.1002682.s003.tif]

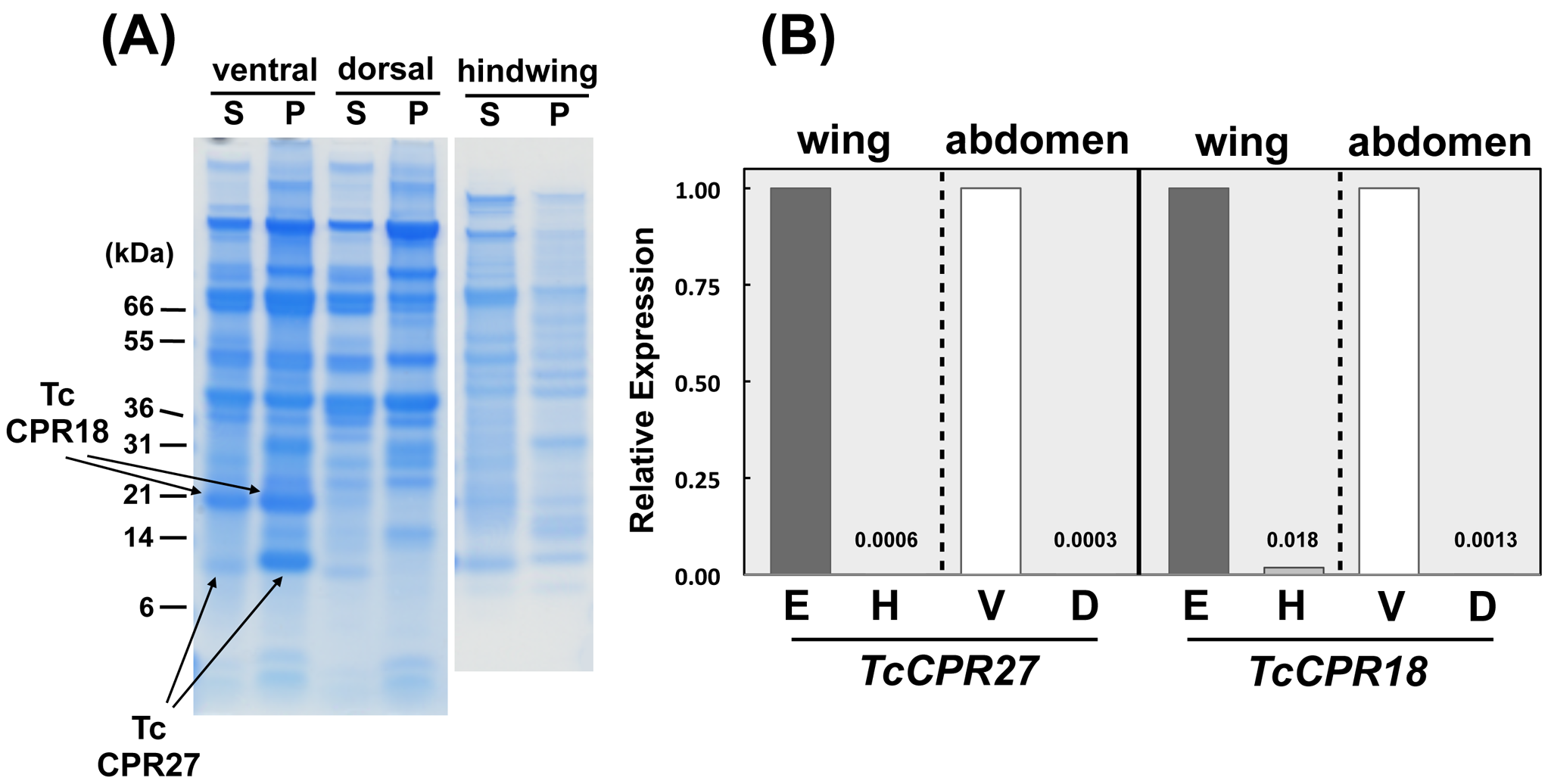

Supplement: Figure S4 — Expression patterns of TcCPR27 and TcCPR18 in the adult ventral vs. dorsal abdominal cuticles and elytra vs. hindwings. (A) The ventral and dorsal abdominal cuticles were dissected from five 0–30 min old adults. TcCRP27 and TcCRP18 were identified by peptide mapping in the ventral abdominal cuticle but not in the dorsal abdominal cuticle. S: PBS homogenate supernatant, P: PBS homogenate pellet. (B) To analyze the transcript levels of TcCPR27 and TcCPR18 in the ventral and dorsal abdomen as well as in the elytra and hindwings, real-time PCR was done using total RNA extracted from tissues of ten pharate adults (5 d-old pupae). Expression levels for TcCPR27 and TcCPR18 are presented relative to the levels of expression in elytra (E) or ventral abdomen (V). The transcript levels of the T. castaneum ribosomal protein S6 (rpS6) were measured to normalize for differences between samples in the concentrations of cDNA templates. H: hindwings, D: dorsal abdominal cuticle. (TIF) [file pgen.1002682.s004.tif]

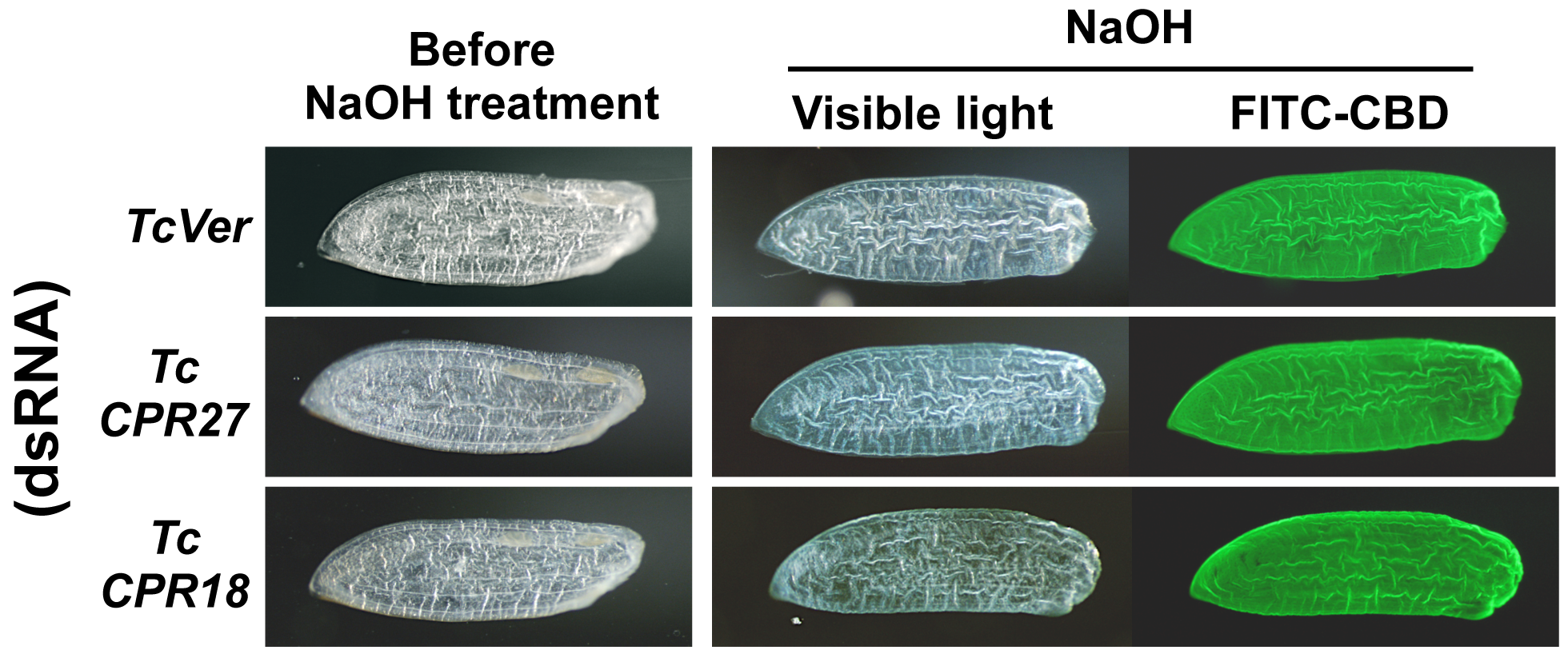

Supplement: Figure S5 — Elytral chitin staining with FITC-CBD. Elytra were removed from pharate adults (5 d-old pupae) that had been injected dsRNA for TcCPR27, TcCPR18 or TcVer (200 ng per insect) at the late larval instar stage. The elytra were incubated with 10 N NaOH at 95°C for 5 h to remove protein, followed by staining with the fluorescein-conjugated chitin-binding domain probe (FITC-CBD, New England BioLabs) [38]. The appearance of the elytra did not differ until after adult eclosion, although dsTcCPR27- and dsTcCPR18-elytra were remarkably soft and fragile compared with dsVer-elytra. The fluorescence was observed using a Leica MZ FLIII fluorescence stereomicroscope equipped with the following filter set: excitation = 480/40 nm, barrier = 510 nm. (TIF) [file pgen.1002682.s005.tif]

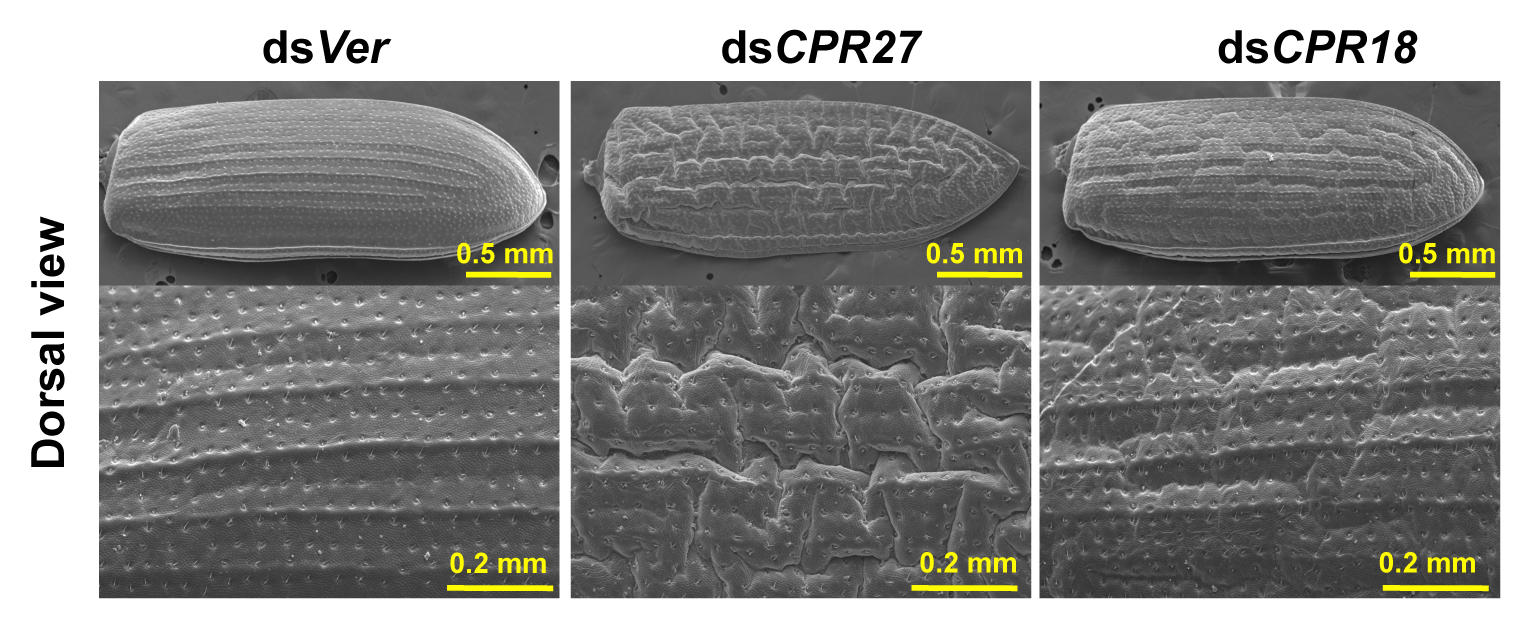

Supplement: Figure S6 — Scanning electron micrographs of TcCPR27- and TcCPR18-deficient elytra. Elytra were dissected from 1 d-old adults that had been injected with dsRNA for TcCPR27, TcCPR18 or TcVer (200 ng per insect) as last instar larvae. The dorsal view of elytra is shown. dsRNA for Ver was injected to serve as a negative control. (TIF) [file pgen.1002682.s006.tif]

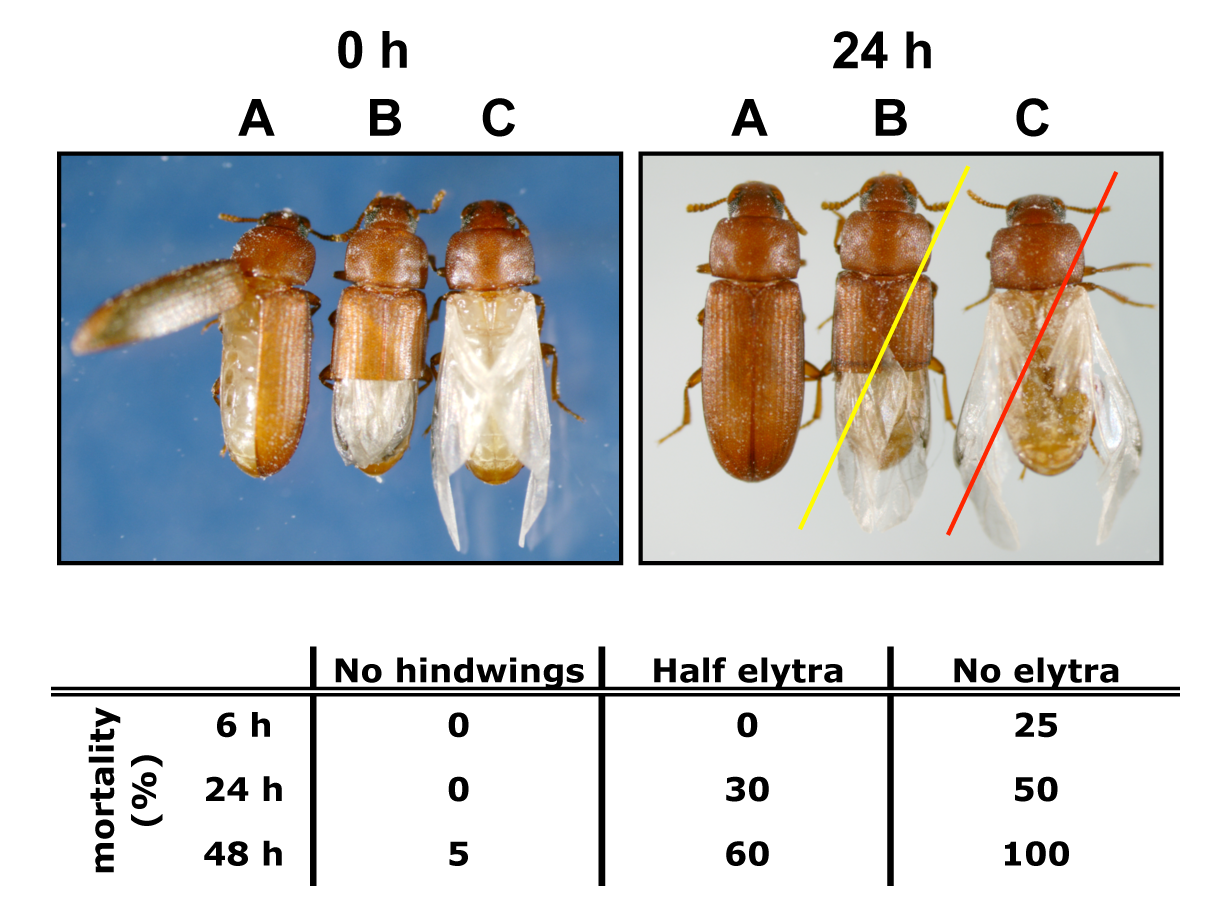

Supplement: Figure S7 — Survival rate after removing elytra or hindwings from mature T. castaneum adults. Elytra or hindwings were removed from mature adults (n = 20), and viability was monitored (insects were reared at 30°C and 50% humidity). A: whole hindwings removed. B: half of distal part of elytra removed. C: whole elytra removed. Loss of an entire hindwing did not affect adult survival, whereas removing elytra resulted in high mortality, probably because of dehydration. Thus, the elytra but not hindwings are essential for T. castaneum adult viability. Yellow and red lines indicate moribund and dead adults, respectively. (TIF) [file pgen.1002682.s007.tif]
