# Supplementary material for: Formation of Rigid, Non-Flight Forewings (Elytra) of a Beetle Requires Two Major Cuticular Proteins
Source: PLoS Genet. 2012 Apr 26;8(4):e1002682. doi: 10.1371/journal.pgen.1002682 (PMC3343089; doi:10.1371/journal.pgen.1002682)
Supplement: Table S1 — Major proteins identified in extracts of unsclerotized elytra. Based on the cDNA sequence, the amino-terminal amino acid residue for both proteins is predicted to be a glutamine (Figure 1). The observed mass of native 10 kDa band was 11,467 Da as determined by MALDI-linear-TOF MS, suggesting that amino-terminus of mature protein of TcCPR27 might be a modified glutamine. Further analysis of MS and MS/MS profiles revealed that the most likely candidate for the amino-terminal residue is pyroglutamic acid. The tryptic peptide of 2143.1 Da from TcCPR27 corresponds to pyro-E20GGEGYGHHHLEEYIDYR37. A similar amino-terminal modification was confirmed in the 20 kDa band identified as TcCPR18 (1451.5 Da peptide, pyro-E20GGGGGEEEYGHHR33). These results suggest that for both proteins, Gln 20 is both deamidated and then dehydrated to form the observed modification. (DOC) [file pgen.1002682.s008.doc]

| **Supplementary Table S1. Major proteins identified in extracts of unsclerotized elytra** | | | | | | |
| --- | --- | --- | --- | --- | --- | --- |
| Gel Band | Protein name | No. of unique peptides | Sequence coverage (%) | Mascot Score (PMF) | Matched peptide sequence | MS/MS  Ion Score |
| 10 kDa | TcCPR27 | 9 | 74 | 120 | Q*GGEGYGHHHLEEYIDYR | 92 |
|  |  |  |  |  | YHYDYNVHDHHTHDFHHQWEHR |  |
|  |  |  |  |  | EVKGEYSLIQPDGR |  |
|  |  |  |  |  | GEYSLIQPDGR | 31 |
|  |  |  |  |  | RTVEYR |  |
|  |  |  |  |  | TVEYR |  |
|  |  |  |  |  | HGADYR |  |
|  |  |  |  |  | IKYEGHSHHGGIGSFGIGGN | 102 |
|  |  |  |  |  | YEGHSHHGGIGSFGIGGN | 96 |
| 20 kDa | TcCPR18 | 16 | 61 | 176 | Q*GGGGGEEEYGHHR | 68 |
|  |  |  |  |  | GTVIGLSR |  |
|  |  |  |  |  | GIEVGR |  |
|  |  |  |  |  | GHYGGGEEER | 53 |
|  |  |  |  |  | GHYGGGEEERGHEER | 64 |
|  |  |  |  |  | GHEER |  |
|  |  |  |  |  | AHPEYHYEYR | 53 |
|  |  |  |  |  | IRDHK |  |
|  |  |  |  |  | TKDYHQK |  |
|  |  |  |  |  | DYHQK |  |
|  |  |  |  |  | HEVR |  |
|  |  |  |  |  | GTYSLLEPDHK | 63 |
|  |  |  |  |  | VVDYTSDK |  |
|  |  |  |  |  | RGFVAR |  |
|  |  |  |  |  | GFVAR |  |
|  |  |  |  |  | VSYKPHY |  |
| * Glutamine was converted to a pyroglutamic acid. | | | | | | |
|  | | | | | | |
