# Supplementary material for: Formation of Rigid, Non-Flight Forewings (Elytra) of a Beetle Requires Two Major Cuticular Proteins
Source: PLoS Genet. 2012 Apr 26;8(4):e1002682. doi: 10.1371/journal.pgen.1002682 (PMC3343089; doi:10.1371/journal.pgen.1002682)
Supplement: Table S2 — Identification of major proteins extracted from elytra of three Tribolium species. (DOC) [file pgen.1002682.s009.doc]

| **Supplementary Table S2.**  **Identification of major proteins extracted from elytra of three *Tribolium* species** | | | | |
| --- | --- | --- | --- | --- |
| Species | Band name | Matched *Tribolium castaneum* protein | Matched peptide sequence | MS/MS  Ion Score |
| *T. brevicornis* | a | TcCPR18 | GTYSLLEPDHK | 29 |
|  | b | TcCPR27 | GEYSLIQPDGR | 37 |
|  | c | TcCPR27 | GEYSLIQPDGR | 33 |
| *T. confusum* | d | TcCPR18 | AHPEYHYEYR | 56 |
|  | e | TcCPR27 | GEYSLIQPDGR | 29 |
| *T. freemani* | f | TcCPR18 | GTYSLLEPDHK | 36 |
|  |  | TcCPR18 | AHPEYHYEYR | 69 |
|  | g | TcCPR18 | GTYSLLEPDHK | 50 |
|  |  | TcCPR18 | AHPEYHYEYR | 57 |
|  | h | TcCPR18 | GEYSLIQPDGR | 37 |
|  |  | TcCPR18 | IKYEGHSHHGGIGSFGIGGN | 73 |
|  |  |  |  |  |
